# Editorial: The innate immune system in rheumatoid arthritis

**DOI:** 10.3389/fimmu.2022.1089522

**Published:** 2022-11-21

**Authors:** Zhu Chen, Javier Leceta, Ali A. Abdul-Sater, Mario Delgado

**Affiliations:** ^1^ Department of Rheumatology and Immunology, The First Affiliated Hospital of University of Science and Technology of China (USTC), Division of Life Sciences and Medicine, University of Science and Technology of China, Hefei, China; ^2^ Department of Cell Biology, Faculty of Biology, Complutense University of Madrid, Madrid, Spain; ^3^ School of Kinesiology and Health Science, Muscle Health Research Centre, York University, Toronto, ON, Canada; ^4^ Department of Immunology and Cell Biology, Institute of Parasitology and Biomedicine Lopez-Neyra (IPBLN-CSIC), Granada, Spain

**Keywords:** arthritis, rheumatoid, innate immunity, inflammation, osteoclast

Rheumatoid arthritis (RA) is a chronic autoimmune-mediated inflammatory disease that affects around 1% of world population. It is the consequence of a failure in self-tolerance mechanisms that facilitate the production of autoantibodies such as rheumatoid factor (RF) and anti-citrullinated protein antibodies (ACPAs) (1). Although dysregulated adaptive immune system as a key player in the pathogenesis of RA have been thoroughly investigated, increasing attention is also being paid to the involvement of the innate immune system in RA (2, 3). Cells of the innate immune system, such as monocytes, macrophages, neutrophils, dendritic cells, and innate lymphoid cells (ILCs), have been implicated in the development, chronicity and resolution phase of RA (2, 3). Hence, understanding how the innate immune system participates in the mechanisms of inflammatory processes as well as bone damage of RA is of great importance.

Activation of the NLRP3 inflammasome and subsequent induction of pro-inflammatory cytokines like IL-1β and IL-18 have been demonstrated in both arthritic animal models and RA patients (4–6). Yin et al. reviewed the current evidence of NLRP3 inflammasome involvement in RA pathogenesis, indicating that inhibition of NLRP3 inflammasome-related signaling pathway could be employed as a potential therapeutic target. Platelets are recognized as innate immune cells and elevated circulating platelet numbers are associated with more severe RA (7). Jiang et al. summarized the latest knowledge on the role of platelet activation in the pathogenesis of RA. Indeed, platelet-based therapeutic targets for RA have been explored (8).

Osteoclasts are the sole bone-resorbing cells that are responsible for the bone erosion in RA. It has been established that FcγR signaling promotes osteoclast differentiation and bone loss in RA, whereas interferon (IFN) γ secreted by immune cells blocks osteoclast activation (9, 10). In this Research Topic, Groetsch et al. investigated the interconnection between the two pathways in regulating osteoclast differentiation in RA. Interestingly, they found that the inhibitory effect of IFNγ on human osteoclast differentiation depends on the osteoclast differentiation stage indicating that IFNγR activation inhibits the formation of osteoclasts in early osteoclast precursors but is enhanced in premature osteoclasts. Furthermore, IFNγR activation on early precursor cells, but not on premature osteoclasts, induces FcγR expression, suggesting a co-regulation of both receptors on osteoclast differentiation, which might reflect their distinct role in different stages of RA.

Periodontitis (PD) has been linked to the development of RA in previous observational studies (11). Yin et al investigated the causal association of PD with RA by a two-sample bidirectional Mendelian randomization (MR) analysis. Surprisingly, their results reveal non-causal association of PD with RA, suggesting that more mechanistic studies are needed to validate the association of PD with RA.

The aryl hydrocarbon receptor (AHR) signaling pathway has been implicated in the regulation of inflammatory diseases including RA (12). Zhang et al explored the association of single nucleotide polymorphisms (SNPs) of AHR signaling pathway genes with RA susceptibility. Despite the absence of significant association between AHR gene polymorphisms and RA susceptibility, they found altered AHR methylation levels were related to the risk of suffering RA.

Collectively, the original research and mini-review articles in this Research Topic cover a series of important aspects in the field of innate immune system in RA.

## Author contributions

ZC drafted the editorial, JL, AA-S and MD reviewed and revised the editorial. All authors contributed to the article and approved the submitted version.

## Conflict of interest

The authors declare that the research was conducted in the absence of any commercial or financial relationships that could be construed as a potential conflict of interest.

## Publisher’s note

All claims expressed in this article are solely those of the authors and do not necessarily represent those of their affiliated organizations, or those of the publisher, the editors and the reviewers. Any product that may be evaluated in this article, or claim that may be made by its manufacturer, is not guaranteed or endorsed by the publisher.
